# Seroprevalence of Dengue Antibodies in Three Urban Settings in Yucatan, Mexico

**DOI:** 10.4269/ajtmh.17-0382

**Published:** 2018-02-19

**Authors:** Norma Pavía-Ruz, Diana Patricia Rojas, Salha Villanueva, Pilar Granja, Angel Balam-May, Ira M. Longini, M. Elizabeth Halloran, Pablo Manrique-Saide, Hector Gómez-Dantés

**Affiliations:** 1 Regional Research Center Hideyo Noguchi, Universidad Autonoma de Yucatan, Merida, Mexico;; 2 Department of Epidemiology, College of Public Health and Health Professions and College of Medicine, University of Florida, Gainesville, Florida;; 3 State Public Health Laboratory, Ministry of Health, Merida, Mexico;; 4 Department of Biostatistics, College of Public Health and Health Professions and College of Medicine, University of Florida, Gainesville, Florida;; 5 Center for Inference and Dynamics of Infectious Diseases, Fred Hutchinson Cancer Research Center, Seattle, Washington;; 6 Department of Biostatistics, University of Washington, Seattle, Washington;; 7 Collaborative Unit for Entomological Bioassays, Universidad Autonoma de Yucatan, Merida, Mexico;; 8 National Institute of Public Health, Cuernavaca, Mexico

## Abstract

Dengue transmission in Mexico has become a major public health problem. Few epidemiological studies have examined the seroprevalence of dengue in Mexico, and recent estimates are needed to better understand dengue transmission dynamics. We conducted a dengue seroprevalence survey among 1,668 individuals including all age groups in three urban settings in Yucatan, Mexico. Children (< 19 years old) were selected randomly from schools. The adults (≥ 19 years old) were selected from healthcare facilities. Participants were asked to provide a venous blood sample and to answer a brief questionnaire with demographic information. Previous exposure to dengue was determined using indirect immunoglobulin G enzyme-linked immunosorbent assay. The overall seroprevalence was 73.6%. The age-specific seroprevalence increased with age, going from 51.4% (95% confidence interval [CI] = 45.0–57.9%) in children ≤ 8 years to 72% (95% CI = 66.3–77.2%) in the 9- to 14-years old. The highest seroprevalence was 83.4% (95% CI = 77–82.2%) in adults greater than 50 years. The seroprevalence in Merida was 68.6% (95% CI = 65–72%), in Progreso 68.7% (95% CI = 64.2–72.8%), and in Ticul 85.3% (95% CI = 81.9–88.3%). Ticul had the highest seroprevalence in all age groups. Logistic regression analysis showed that age and city of residence were associated with greater risk of prior dengue exposure. The results highlight the level of past exposure to dengue virus including young children. Similar studies should be conducted elsewhere in Mexico and other endemic countries to better understand the transmission dynamics of dengue.

## INTRODUCTION

Dengue is a global public health problem affecting more than 100 countries, with approximately three billion people at risk and an estimated 390 million dengue infections annually worldwide.^1^ Dengue is one of the vector-borne viral diseases with high impact throughout the Americas with an average of 1.8 million of dengue cases reported during the current decade.^2^ Brazil, Colombia, and Mexico account for the largest burden of disease in the region.^3,4^

Although dengue epidemics have increased significantly worldwide,^1,5^ global estimates of dengue distribution and disease burden remain imprecise in most dengue endemic areas.^6,7^ The real burden of dengue is unknown as most dengue infections are asymptomatic, misdiagnosed, or not reported.^6^ Some of the main issues in dengue surveillance include the lack of standardized reporting procedures, variable diagnostic laboratory capacity in traditional surveillance systems, along with the absence of reporting from the private health sector, which in our study population represents an important proportion of all healthcare providers.^8^ An important, but underutilized tool in some countries is population-level dengue antibody surveys. Seroprevalence surveys are invaluable in identifying the burden of both symptomatic and asymptomatic infections and quantifying infection prevalence and incidence in different epidemiological settings.^9–13^

Although dengue virus (DENV) has been circulating in Mexico since the late 1970’s, recently there have been larger and more frequent dengue outbreaks with an increasing proportion of severe cases being reported to the national epidemiological surveillance system. In addition, co-circulation of the four serotypes of DENV is being detected more often since 2010 compared with the previous decades.^14^ Dengue surveillance in Mexico is conducted passively through the report of suspected dengue cases by the doctors in the healthcare facilities. As a result, only a small fraction of symptomatic cases are diagnosed and reported through the surveillance system,^15^ and hospital records only partially report the incidence of severe dengue cases.^16^ These reported cases are likely a small proportion of the total dengue burden, especially in economically vulnerable communities that may not be seeking healthcare, and therefore, are experiencing undetected cases of dengue. In addition, there is scant information regarding the extent of transmission in cities and suburban areas in Mexico. There is also limited information regarding the proportion of asymptomatic and mild infections in the population at large, and potential misdiagnosis of dengue as other endemic infections (leptospirosis,^17^ rickettiosis,^18^ and the recently introduced chikungunya virus [2015]^19^ and Zika virus [2016]^20^) because of limited diagnostics.^21,22^ Despite the known increase in the reported number of dengue and severe dengue cases,^23^ there are few seroprevalence surveys in Mexico. More information is needed to understand the epidemiology of the disease in Mexico over the last three decades. One of the regions of concern with regard to dengue transmission and outbreaks in Mexico is the state of Yucatan. Dengue transmission has been documented in Yucatan since 1979, and in the past two decades, there have been large dengue outbreaks and annual co-circulation of at least two serotypes increasing the severity of dengue in this area.^23^ During the early 1990s, the circulation of DENV serotypes DENV1 and DENV4 was detected, but DENV3 was the most often isolated serotype in Yucatan. In the early 2000s, most of the outbreaks were due to DENV2. Since 2006, DENV2 has been co-circulating with DENV1 and DENV4.^23^

In this article, we present the results of a cross-sectional dengue serosurvey conducted in three cities in the state of Yucatan in 2014, as part of a series of baseline studies necessary to better understand dengue transmission dynamics in three different settings in Yucatan, Mexico. This baseline dengue seroprevalence information of at risk populations in Yucatan will allow for the assessment and modeling of the potential impact of a dengue vaccine^9^ and will function as a benchmark to evaluate the effectiveness of different intervention strategies, such as vector control and vaccine introduction, among others.^24–29^

## METHODS

### Study settings.

The state of Yucatan is located in the southeastern peninsula of Mexico and borders the Gulf of Mexico and the Caribbean Sea. We chose to conduct our study in three cities that exemplify different levels of transmission based on historical epidemiological surveillance data. We chose a high transmission setting (Merida), a medium transmission setting (Ticul), and a low transmission setting (Progreso).^30^ Merida, the capital city of Yucatan, is the largest urban center in the region with a population of 814,000 inhabitants. The weather is warm throughout the year (mean annual temperature is 25.9°C, ranges from 19.5 to 33.6), and the rainy season occurs from June to October with an annual precipitation of 1,050 mm. Progreso is the main seaport in the state, is located 32 km north of Merida, and has a population of 54,000 inhabitants. The weather in Progreso is similar to Merida. Progreso is a destination for national and international tourists—its population increases during the weekends and holidays (July–August). Ticul is a rural town located 82 km south of Merida with 34,000 inhabitants, and the weather is similar to Merida.

### Selection of survey population.

Our objective was to estimate the overall level of exposure to dengue (as measured by prevalence of antibody to dengue) in populations within the three study sites in Yucatan, with an emphasis on the younger population (< 19 year olds) that could be possibly targeted for vaccination. The desired survey sample size was estimated based on 50% prevalence and a 95% confidence interval (95% CI), which suggested a sample size of 1,307 individuals for our study. We increased the sample size to 1,700 to increase the number of children (< 19 year olds) in the study (they comprised half of the samples) and distributed it according to the population size in the three settings, with a higher proportion of samples coming from Merida, followed by Progreso and Ticul.

The sampling scheme included two different approaches. The first one was a school-based survey with a random selection of schools in the three cities followed by a random selection of children (5–19 years old) in the selected elementary, middle, and high schools of Merida, Ticul, and Progreso. We selected eight schools from Merida (five were elementary schools), six schools from Progreso (four were elementary schools), and five schools from Ticul (three were elementary schools). Prior agreements and an explanation of the study aims were made with the local ministries of Health and Education of Yucatan. We requested the lists of students by grade to obtain ages of the participants and their information. We did a stratified randomization by grade and school to have a proportional sample in each age group. We excluded participants whose siblings were previously randomized and had provided signed informed consent. In the city of Merida, we confined the study to schools in the central metropolitan area. Blood samples were obtained from students at school after their parents signed the informed consent forms. The results of the serosurvey were delivered personally to the parents of the participating children.

The adults (≥ 19 years old) were stratified by age and randomly selected from people attending public primary healthcare centers in Merida, Progreso, and Ticul. We included individuals who did not have signs of acute febrile illness, who were requesting a health certificate or were under clinical follow-up for a noncommunicable disease. Because the survey sample was not proportional to age and sex of the population in the locality, weights were calibrated to reproduce the age distribution of the population in each locality and reduce sample bias. Blood samples were obtained after the study participants signed the informed consent forms. For the adult study participants, their serosurvey results were reported to them by telephone or home visits.

### Study procedures.

Participants were asked to provide a 5-mL venous blood sample and to complete a brief questionnaire with basic demographic information. Blood samples were collected in anticoagulant-free Vacutainer tubes by trained and certified health personnel, centrifuged within 1.3 hours of collection, and transported to the state laboratory. Samples were stored at −70°C ± 5°C until the serological testing was done. Serum samples were obtained from individuals and tested for prior dengue exposure in the state public health laboratory (LESPRE) of the Ministry of Health. Prior exposure to dengue was determined by testing for immunoglobulin G (IgG) antibody to dengue using Panbio IgG indirect enzyme-linked immunosorbent assay (ELISA). Standard cutoff points were used for defining positive (≥ 12 Panbio units) and negative samples (< 9 Panbio units). Samples that had reading in between these cutoff thresholds were classified as “indeterminate.”

### Ethical review.

This study was approved by the institutional review boards at Fred Hutchinson Cancer Research Center and the General Hospital Agustín O’Horan of Yucatan. Written consent was obtained from all adult participants (≥ 19 years old) after providing them with a detailed explanation of the study and procedures. Parents/guardians of all child participants (< 19 years old) were asked to provide written consent on their behalf.

### Statistical analysis.

Descriptive analysis of the demographic variables and a logistic regression model analysis were carried out using R (version 3.2.2).^31^ Age was grouped into five categories: ≤ 8, 9–14, 15–19, 20–49, and ≥ 50 years. We estimated the age-specific dengue antibody prevalence and fit a logistic regression model of the variables independently associated with dengue seropositivity. The baseline category for the logistic regression was to be dengue seronegative. The relationship was estimated as the odds ratio. The independent variables considered were age (≤ 8 years old considered as the reference group), gender (male as the reference group), place of residence (Merida as reference), previous history of dengue (not having previous history of dengue as reference), and previous dengue confirmation (no previous confirmation of dengue as reference).

## RESULTS

### Description of the study population.

**Figure 1.**
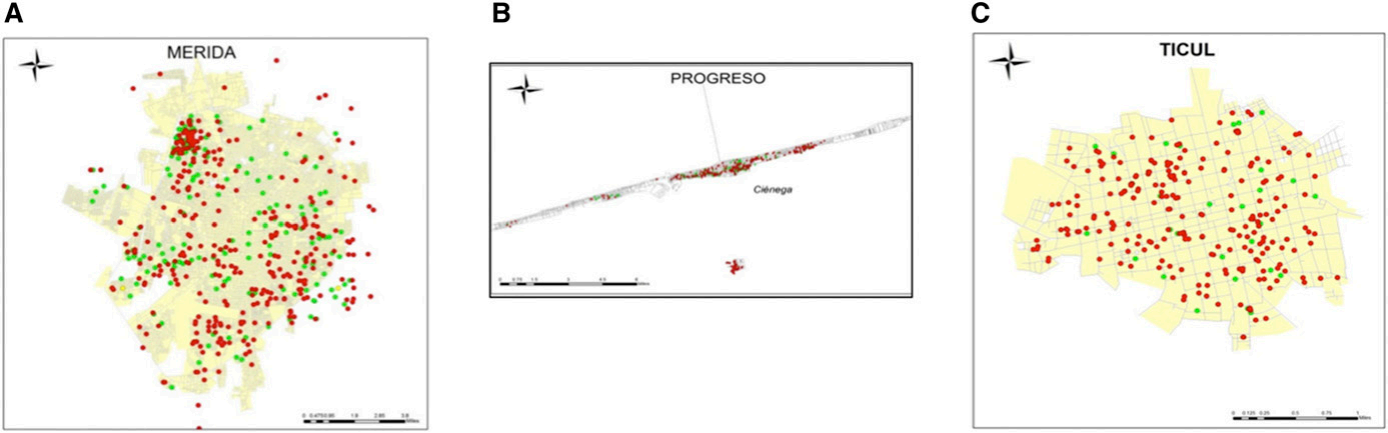
Geographic location of the participants in the seroprevalence study. (**A**) Geographic location of participants from Merida, (**B**) Geographic location of participants from Progreso, and (**C**) Geographic location of participants from Ticul. The red dots show dengue seropositive individuals and green the seronegatives. This figure appears in color at www.ajtmh.org.

From January through June 2014, a total of 1,731 serum samples were collected in the cities of Merida, Progreso, and Ticul. Most of the samples (43%) were collected in Merida (748), 27% in Progreso (472), and 30% in Ticul (511). Most of the samples were obtained from healthcare centers (56%), whereas 24% were collected from elementary schools and 20% from middle and high schools. We analyzed 1,667 blood samples from people from the three cities: Merida (700), Progreso (469), and Ticul (498). The collected samples were from people distributed widely through the neighborhoods in the selected cities (Figure 1). After processing the samples, 3.6% (63/1,731) had indeterminate results for the IgG ELISA and were excluded from our analyses.

The mean age in the overall study population was 25.8 years (standard deviation = 18.04), with no significant difference in the mean population age between the three surveyed cities (*P* = 0.172). The population was evenly distributed by age group, except for the 9 to 14 and 15 to 19 year-old age groups in Progreso as compared with Merida and Ticul (Table 1). Most of the participants were female (62%), which is higher than the proportion of females (51%) in Yucatan. We found significant differences in gender distribution between cities (with some populations having more females sampled; *P* < 0.01).

**Table 1:** Demographic characteristics of the study population in Yucatan, Mexico (*N* = 1,667)

	Merida		Progreso		Ticul		Total	
	*n*	Percent	*n*	Percent	*N*	Percent	*n*	Percent
Age (years)								
≤ 8	101	14.5	71	15.1	69	13.8	241	14.5
9–14	90	12.8	110	23.4	75	15.0	275	16.5
15–19	128	18.3	64	13.6	93	18.7	285	17.0
20–49	292	41.7	175	37.5	188	37.8	655	39.3
50+	89	12.7	49	10.4	73	14.7	211	12.7
Sex								
Male	291	41.5	157	33.1	184	36.9	632	37.9
Female	409	58.5	312	66.9	314	63.1	1,035	62.1
Born in Yucatan								
Yes	624	89.1	440	93.8	487	97.8	1,551	94.0
No	76	10.9	29	6.2	11	2.2	116	7.0
History of previous dengue								
Yes	60	8.6	16	3.4	10	2.0	86	5.2
No	640	91.4	453	96.6	488	98.0	1,581	94.8
Previous confirmation of dengue								
Yes	27	3.9	12	2.6	8	1.6	47	2.8
No	673	96.1	457	97.4	490	98.4	1,620	97.2

The majority of the study population (94%) was born in Yucatan, with Ticul having a significantly higher proportion (98%) born in Yucatan as compared with Progreso (93.8%) and Merida (89.1%) (Table 1). This may be because Ticul is a rural town, and the inhabitants are less likely to leave the state (observation by field team in Ticul).

A small proportion of the participants (5%) mentioned having previous history of dengue. This proportion was higher in Merida (8.6%), followed by Progreso (3.4%) and Ticul (2%). We also found that a small proportion (2.8%) of the study population recalled having previous laboratory confirmation of dengue, and most of these confirmed cases were from Merida (3.9%), potentially because as the capital of Yucatan, accessibility to health care is highest in Merida as compared with the other two cities. The proportion of laboratory confirmation was higher in the adult population.

### Dengue seroprevalence.

**Figure 2.**
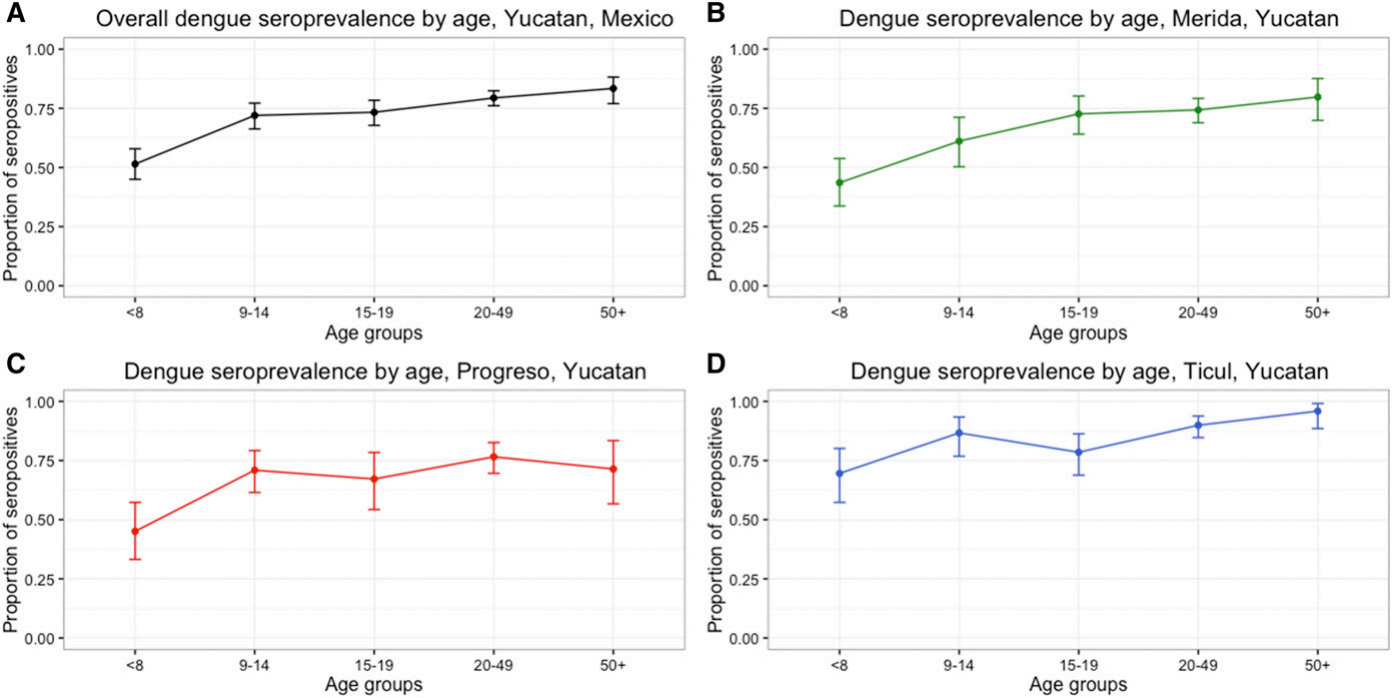
Dengue seroprevalence to indirect immunoglobulin G (IgG) by age group and city, Yucatan, Mexico 2014. (**A**) Overall dengue seroprevalence; (**B**) Dengue seroprevalence, Merida; (**C**) Dengue seroprevalence, Progreso; and (**D**) Dengue seroprevalence, Ticul. This figure appears in color at www.ajtmh.org.

The overall estimated dengue antibody prevalence in Yucatan was 73.6% (95% CI = 71.4–75.7%), indicating previous dengue exposure in most of the population. Dengue seroprevalence in the Yucatan increased with age in all three study settings (Figure 2).

Overall, children ≤ 8 years old had the lowest seroprevalence (51.4% [95% CI = 45–57.9%]) and adults ≥ 50 years old had the highest seroprevalence (83.4% [95% CI = 77–88.2%]) (Figure 2A).

The seroprevalence was highest in the Ticul population (85.3% [95% CI = 81.9–88.3%]) and was similar in the other two cities (Merida: 68.6% [95% CI = 65–72%] and Progreso: 68.7% [95% CI = 64.2–72.8%]). The estimated dengue seroprevalence in Merida ranged from 43.6% (95% CI = 33.7–53.8%) in ≤ 8-year-old children to 79.8% (95% CI = 69.9–87.6%) in ≥ 50-year-old adults (Figure 2B). In Progreso, the seroprevalence ranged from 45.1% (95% CI = 33.2–57.3%) in the younger age group (≤ 8-year olds) to 71.4% (95% CI = 56.7–83.4%) in the ≥ 50-year-old (Figure 2C) group. Ticul had the highest dengue seroprevalence in all age groups compared with the other two cities; it ranged from 69.6% (95% CI = 57.3–80.1%) in ≤ 8-year olds to 95.9% (95% CI = 88.5–99.1%) in ≥ 50-year olds (Figure 2D).

Our logistic regression model showed that there were significant relationships between being positive for dengue antibody and increased age. The reference group for age was the ≤ 8 years old group. The 9–14 year old group had 2.43 (95% CI = 1.69–3.50) times the odds of being seropositive compared with the reference group (Table 2). The ≥ 50 years group had 4.74 (95% CI = 3.08–7.46) times the odds of being seropositive compared with the reference group. Females had 1.27 (95% CI = 1.02–1.59) times the odds of being seropositive compared with males. Participants from Ticul had 2.67 (95% CI = 1.99–3.60) times the odds of being seropositive compared with the participants from Merida.

**Table 2:** Logistic regression model of risk factors associated with dengue seropositivity in Yucatan, Mexico

Variables	Total (*N* = 1,667)	Seropositives (*N* = 1,227)	Seronegatives (*N* = 440)	Odds ratio	95% CI
Age (years)					
< 8	241 (14.5)	124 (10.1)	117 (26.6)	Ref	Ref
9–14	275 (16.5)	198 (16.1)	77 (17.5)	2.43	1.69–3.50
15–19	285 (17.0)	209 (17.0)	76 (17.2)	2.59	1.81–3.75
20–49	655 (39.3)	520 (42.4)	135 (30.7)	2.63	2.65–4.99
50+	211 (12.7)	176 (14.3)	35 (8.0)	4.74	3.08–7.46
Sex					
Male	632 (37.9)	445 (36.3)	184 (42.0)	Ref	Ref
Female	1,035 (62.1)	781 (63.7)	254 (58.0)	1.27	1.02–1.59
City					
Merida	700 (42.0)	480 (39.1)	220 (50.0)	Ref	Ref
Progreso	469 (28.1)	322 (26.2)	147 (33.4)	1.01	0.78–1.29
Ticul	498 (29.9)	425 (34.7)	73 (16.6)	2.67	1.99–3.60
Born in Yucatan					
No	116 (6.9)	77 (6.3)	39 (8.9)	Ref	Ref
Yes	1,551 (93.1)	1,150 (93.7)	401 (91.1)	1.45	0.96–2.16
History of previous dengue					
No	1,581 (94.9)	1,163 (94.8)	418 (95.2)	Ref	Ref
Yes	86 (5.1)	64 (5.2)	21 (4.8)	1.09	0.67–1.86
Previous confirmation of dengue					
No	1,620 (97.2)	1,190 (97.0)	428 (97.3)	Ref	Ref
Yes	47 (2.8)	37 (3.0)	12 (2.7)	1.25	0.64–2.70

CI = confidence interval.

The characteristics of: being born in Yucatan, self-reported history of previous infections with dengue and self-reported previous confirmation of dengue were not associated with seropositivity (Table 2).

## DISCUSSION

Our study found an overall dengue antibody prevalence of 73.6% in our surveyed cities in Yucatan, consistent with previous studies in this area. In contrast to previous studies, we found that women had a higher dengue antibody prevalence than males. In addition, we found that Ticul, a city previously classified as an area with a medium level of dengue transmission, had the highest prevalence of previously exposed individuals (85.3% versus ∼68% in the other surveyed cities), and this observation held when comparing all the surveyed age groups. More than 70% of children in the age group between 9 and 14 years had been exposed to dengue, which has important implications to help targeting dengue vaccines and maximizing future vaccine impact.

Our survey is the most recent cross-sectional study in the state of Yucatan (first survey was in 1985), and our overall prevalence was similar to other dengue serosurveys carried out in Mexico and the Yucatan.^32^ In 1985, the prevalence of anti-dengue antibodies in the urban population of Yucatan was 72.5%, very similar to our overall seroprevalence estimates.^33^ Another study in a cohort of school children from 8 to 14 years old (1987–1988) including urban and rural localities of Merida reported an overall dengue seroprevalence of 56.3% in children living in urban areas compared with 63.7% in children living in rural areas.^34^ Comparing these results from almost 20 years ago with our current prevalence estimate for Merida (68.8%), one can conclude that Merida has a stable (endemic) dengue transmission pattern. More recent studies have found an overall dengue antibody prevalence in Yucatan of 59.9% and 81.5% in 1996 and 2006 respectively.^35^ When comparing our results with other Latin American countries with endemic dengue, our estimates were similar to those from Venezuela and Brazil but lower than those from Nicaragua.^22,36–38^

In our study, women were more likely to be antibody positive compared with men. Previously, cases reported to the surveillance system indicated that there was a similar exposure pattern of women (51%) and men (49%) to dengue.^34^ Our sample population was 59% female, which may be why we saw this difference. However, the prevalence rates from the public health surveillance system only captures symptomatic cases that attend a healthcare facility, which likely greatly underestimates the overall level of dengue exposure. Therefore, our observed pattern may be related to dengue transmission patterns—there may be more dengue transmission within a household setting, and women in Yucatan are more likely to stay at home compared with men. Additional community or population-based antibody surveys are necessary to better clarify this relationship of dengue exposure and sex.

We found that there was a statistically significant relationship between age and exposure to dengue, with older age classes showing higher levels of exposure than younger age classes. The other variables tested were not significantly associated with seropositivity in the study population. In our study, around half of the younger children (≤ 8 years old) were antibody positive to dengue, whereas more than 80% of adults > 50 years old were positive for dengue antibody. The overall dengue antibody prevalence in children and adolescents 9–14 years old was 72%. This is a relevant finding given the World Health Organization (WHO) recommendations for introduction of the first dengue vaccine that is also licensed in Mexico (CYD-TDV-Dengvaxia).^39^ This vaccine was licensed targeting the population from 9 to 45 years old. The WHO recommends introducing the dengue vaccine (Dengvaxia) only in geographic settings where epidemiological data suggest a high burden of disease (seroprevalence ≥ 70%) in the populations to be targeted for vaccination to maximize public health impact and cost-effectiveness.^39,40^ This high burden can be objectively measured by dengue seroprevalence surveys.

Ticul had the highest seroprevalence in all age groups. This may be a result of a recent DENV1 outbreak in 2013 there. The similar prevalence estimates of Merida and Progreso are associated with an endemic transmission pattern. The varied exposure patterns among the populations from the three surveyed urban areas indicate that there is heterogeneous dengue transmission across Yucatan.

One of the main limitations of this study is the lack of dengue serotype–specific prevalence information and the reliance on IgG ELISA without neutralizing assays for confirmation or for serotyping. Identifying serotypes could help better understand the intensity of transmission and establish whether the overall population or specific age groups have been exposed to and the level of exposure to one or more serotypes. This serosurvey was conducted before the introduction of Zika virus to Mexico, so we are confident that the ELISA results were not cross-reacting with other flaviviruses. Our sampling strategy focused on enrolling adults in the healthcare centers and intentionally oversampled the population < 19 years old because this particular group is the potential target vaccination group in Mexico, so it is not a true random sample of the population. As with all self-reported health histories, there was likely some recall bias when interviewing people about having had dengue. As this survey was conducted in school children and adults attending public health services provided by the Ministry of Health, these individuals likely are from households of lower-and middle socioeconomic status.

As established by the Mexican Dengue Expert Group, it is essential to develop an evidence-based proactive strategy that provides evidence to decision makers for the introduction of dengue vaccines.^41^ These data should include results from clinical trials, epidemiological studies, and better burden of disease estimates to create a sustainable immunization program with all the resources required for the adoption of the new vaccine and future evaluation of its impact and effectiveness.^41^ Our survey provides evidence regarding the serological status of potential target populations in a highly endemic region of the country. Population-based serosurveys, similar to ours, are extremely important to identify age groups with higher dengue risk and to best target interventions.^10,11,27,42–44^ Similar dengue serosurveys should be pursued elsewhere in Mexico and other endemic countries to better understand the transmission dynamics of dengue and to help evaluate and model the impact of the vaccines and other interventions already available and in development.^9,29,44–49^
